# Predicting the Risk of Cardiovascular Diseases in the Elderly Based on Clinical Data and Heart Rate Variability Using Machine Learning

**DOI:** 10.3390/jcm15135141

**Published:** 2026-07-01

**Authors:** Kuat Abzaliyev, Akbota Bugibayeva, Symbat Abzaliyeva, Gulsim Akhmetova, Gulzira Balkanay, Aliya Omarbayeva, Saken Anartayev, Nazima Zarubekova, Madina Suleimenova

**Affiliations:** 1 Department of Big Data and Artificial Intelligence, Faculty of Information Technology, Al-Farabi Kazakh National University, Almaty 050040, Kazakhstan; kuatabzaliev1961@gmail.com (K.A.); abzaliyeva.symbat@gmail.com (S.A.); 2 Department of Postgraduate Education, Kazakhstan’s Medical University “Kazakhstan School of Public Health”, Almaty 050040, Kazakhstan; 3 Department of Emergency and Urgent Medical Care, S.D. Asfendiyarov Kazakh National Medical University, Almaty 050040, Kazakhstan; akhmetova.g@kaznmu.kz (G.A.); gulzira_b@mail.ru (G.B.); zarubekova@mail.ru (N.Z.); 4 Department of Internal Medicine, Faculty of Medicine and Health Care, Al-Farabi Kazakh National University, Almaty 050040, Kazakhstan; aliyaomb@gmail.com; 5 Department of Interventional Cardiology, Arrhythmology and Angiosurgery, City Clinical Hospital 7, Almaty 050006, Kazakhstan; saken_anartaev@mail.com; 6 Department of Cardiology, S.D. Asfendiyarov Kazakh National Medical University, Almaty 050040, Kazakhstan; 7 Department of Information Systems, International Information Technology University, Almaty 050040, Kazakhstan

**Keywords:** cardiovascular disease, heart rate variability, predicting, photoplethysmography, machine learning

## Abstract

Cardiovascular diseases (CVDs) remain the leading cause of morbidity and mortality in the elderly worldwide. Over the past two decades, there has been a wealth of evidence of a close relationship between autonomic nervous system activity and cardiovascular mortality, including sudden cardiac death. Heart rate variability (HRV), derived from photoplethysmographic (PPG) signals, is increasingly recognized as a promising non-invasive digital marker for evaluating autonomic nervous system function and stratifying CVD risk. The application of machine learning algorithms to PPG-derived HRV analysis offers a promising approach for improving CVD risk stratification and facilitating the development of personalized medicine strategies. **Background/Objectives**: To evaluate the potential of heart rate variability indicators in predicting the risk of developing CVD in individuals aged 65 years and older. **Methods**: The study involved individuals aged 65 years and older, divided into two groups: those with a risk of developing CVD (*n* = 54) and those without risk (*n* = 46). The first stage included a questionnaire as well as anthropometric and hemodynamic measurements. At the second stage, a PPG was performed using the Eldar computer photoplethysmograph and Eldar-Vario software, followed by an analysis of time-domain and spectral HRV parameters. Statistical data analysis was conducted using the SPSS Statistics 22.0 software package, focusing on the evaluation of associations between HRV indicators and the presence of CVD. Interpretable machine learning models were developed using logistic regression and a random forest algorithm within a nested cross-validation framework. In addition to the discriminatory characteristics, Brier score, LogLoss, calibration analysis, error matrices, permutation importance, and SHAP interpretation were analyzed in the study. **Results**: In patients with cardiovascular diseases, a statistically significant decrease in heart rate variability was revealed: SDNN by 2 times (26 [Q1–Q3: 15, 35] ms), pNN50 by 3.5 times (4 [3, 5]%), TINN by 5 times (31 [20, 51] ms), and HRV by 2.5 times (6 [4, 8.7]). In addition, a decrease was seen in the spectral components of VLF by one-fold (2450 [Q1–Q3: 2450, 4500] ms^2^), LF by four-fold (750 [750, 1500] ms^2^) and HF by five-fold (450 [450, 750] ms^2^) (*p* < 0.05). At the same time, there was a significant increase in the VLF/HF and LF/HF ratios, which indicates a predominance of sympathetic activity. According to the results of the correlation analysis, statistically significant associations of HRV indicators with age, physical activity level, body mass index and systolic blood pressure were revealed. The results of machine learning also revealed the association of HRV with arterial hypertension, physical activity and BMI. The best final results were demonstrated by a random forest model with a combined set of clinical and HRV signs of HF and RMSSD (ROC-AUC was 0.9988). The signs of heart rate variability obtained by photoplethysmography demonstrated additional prognostic value in relation to clinical signs. PPG-derived HRV features demonstrated additional discriminatory value for cardiovascular risk stratification. **Conclusions**: The obtained data demonstrate a close association between the risk of developing cardiovascular disease and autonomic nervous system dysfunction. The decrease in heart rate variability is most pronounced in elderly individuals with existing cardiovascular disease and can be considered a potential tool for developing diagnostic, prognostic, and risk stratification strategies. The use of machine learning demonstrated that heart rate variability features obtained using photoplethysmography improve diagnostic prognostication and classification of cardiovascular diseases compared to models based solely on clinical data.

## 1. Introduction

Currently, despite the presence of a wide range of preventive, diagnostic and therapeutic measures, the pathology of the cardiovascular system (CVS) occupies a leading position among the causes of mortality. Early and accurate assessment of an individual’s cardiovascular disease risk is essential for effective primary prevention, enabling the timely initiation of risk-modifying interventions and optimizing the allocation of screening resources [1,2,3].

Key CVD risk factors include advanced age, male gender, lifestyle habits, metabolic disorders, and biomarkers such as elevated C-reactive protein (CRP) and homocysteine [4,5,6]. Additionally, autonomic dysfunction characterized by sympathetic overactivity and elevated resting heart rate (HR) serves as a critical predictor of CVD development, most often expressed in increased activity of the sympathetic division of the autonomic nervous system and an increase in resting heart rate (HR) [7,8].

A literature review by Abdülmelik Birgün and co-authors (2025) showed the multifaceted role of autonomic functions in the health of the CVS and the development of diseases, characterized by increased sympathetic activity and decreased parasympathetic regulation, as well as the effect on vascular remodeling and increased arterial wall stiffness, increasing the risk of developing CVD [8]. One of the methods for assessing the activity of the cardiovascular and autonomic nervous systems is the analysis of heart rate variability (HRV) [8,9].

A study by J. Wang et al. (2025) showed that dysfunction of the autonomic nervous system affects HRV indicators; the study revealed a link between a decrease in SDNN and increased arterial stiffness (odds ratio = 0.97; 95% CI = 0.95~0.99) in patients with hypertension, especially in those under 65 years of age (*p* < 0.05) [10].

Currently, the study of these indicators is carried out using photoplethysmography (PPG). Due to its low cost, ease of use, and the possibility of long-term monitoring in outpatient settings, PPG is of great importance for mass screening and early diagnosis of latent vascular dysfunction [9,10,11,12]. Researcher Kristjan Pilt conducted an in vitro study of the effects of blood flow profiles on the signals of reflective PPG; according to preliminary data, he suggested that PPG could become the basis for new technologies for assessing the blood flow profile in arteries. In another study, Kristjan Pilt and co-authors (2024) analyzed arterial stiffness according to PPG data in 51 patients without CVD; the results showed that the stiffness index obtained using PPG correlated with the aortic augmentation index (r = 0.79–0.83) [11,12].

Since 2025, scientists have studied PPG indicators as predictors of the risk of developing CVD; for example, Wei-Hung Weng and co-authors (2024) predicted the risk of CVD using PPG and deep learning, and the results showed that clinical and demographic data provide statistically significant prognostic information about the risk of CVD (*p* < 0.001) [13].

There is limited data on how changes in one indicator affect another over the long term. There are a number of problems that need to be solved in clinical practice, such as the interpretation of PPG signals in populations with different physiological characteristics and the relationship between HRV changes and arterial stiffness, taking into account age-related changes, technical issues that have not been resolved, the lack of algorithms for interpreting signals, and promising real-time clinical research [14,15,16,17].

Currently, the use of artificial intelligence in medicine is showing steady growth and significant scientific and practical progress. The integration of machine learning and deep learning methods in the analysis of PPG signals allows us to identify informative biomarkers such as arterial stiffness, HRV, vascular age and other indicators reflecting subclinical vascular disorders and to conduct predictive assessment of cardiovascular events, contributing to a more accurate stratification of patients according to the degree of risk of CVD. Recent studies (Abdullah and Kristoffersson, 2023; Bruno Ferraz 2023, Weng et al., 2024; Barthels et al., 2024; He, B 2024; Nie et al., 2025; Shujuan Liu 2025, etc.) confirm the high potential of these approaches for predicting CVD using a machine algorithm [13,18,19,20,21,22,23].

The purpose of this study is to evaluate the potential of heart rate variability indicators as a method for predicting the risk of developing CVD in individuals aged 65 years and older.

Since this reflects the results of a cross-sectional phase, the term “risk prediction” within this framework is defined as diagnostic prediction models in accordance with the international TRIPOD guidelines. This refers to estimating the mathematical probability of a disease’s presence at the current moment using machine learning classification algorithms [24].

## 2. Materials and Methods

This study is a fragment of the scientific project “Development of markers and diagnostic algorithms for the detection and prevention of premature aging of the cardiovascular system” of the Ministry of Science and Higher Education of the Republic of Kazakhstan, state registration No. AP19677754. To achieve the purpose of this study, a single-stage cross-sectional study was conducted among older adults with and without cardiovascular diseases risk factors. The sample size (*n* = 100) was determined by the established inclusion criteria and the specific timeline of the study protocol. This scientific work represents a pilot study aimed at the primary validation of diagnostic prediction models and assessment of the informative value of HRV metrics under controlled conditions. The study included 100 older adults aged 65 years and older, comprising 40 (40%) men and 60 (60%) women, who were recruited from outpatient clinics and medical centers in Almaty.

Inclusion criteria: individuals aged 65 years and older, both with and without cardiovascular disease risk, who provided written informed consent to participate in the study.

Exclusion criteria: HIV infection, known tuberculosis, acute infectious diseases within 3 months prior to inclusion, mental illnesses that limited adequate cooperation, diagnosed allergic reaction of any type, refusal to participate in the study.

All participants in the study received complete, reliable and objective information about the goals, objectives, methods and possible risks of the study. Before being included in the study, all participants signed an informed voluntary consent to participate in accordance with the World Medical Association Declaration of Helsinki (1964, with subsequent additions and revisions). Patients’ participation in the study was voluntary, and participants had the right to withdraw at any stage without any consequences. This study was approved by the Local Ethical Committee of Al Farabi Kazakh National University (IRB00010790, approval number IRB-A515 from 9 November 2023). The confidentiality of the personal data of the study participants was fully respected.

This study included three stages. The first stage consisted of a questionnaire, an anthropometric study (height, weight, waist circumference, BP, HR, pulse pressure), and determination of total cholesterol and GFR as well as cardiac echocardiography data-ejection fraction. The survey included data on age, gender, medical history of cardiovascular diseases, chronic diseases, medical history, major behavioral risk factors (smoking, alcohol consumption, sedentary lifestyle), and heredity.

At the second stage, a PPG assessment was performed using the Eldar computerized photoplethysmograph, developed and manufactured by Engineering and Medical Center “New Devices” (Samara, Russia) and specialized Eldar-Vario, Version 1.0 software. PPG signals were recorded using a finger-mounted optical sensor attached to the distal phalanx of the index finger. Measurements were conducted in a quiet environment at a room temperature of 22–24 °C, with participants in the supine position. Prior to date acquisition, factors known to affect vascular tone, including physical activity, smoking, caffeine consumption, and psychoemotional stress, were excluded. For HRV analysis, a consecutive series of pulse-to-pulse (NN) intervals was recorded. Time-domain and frequency-domain HRV analyses were performed using a fixed-duration short-term recording of 5 min (300 s), with date acquisition and processing conducted in real time. The noise immunity of the estimates and date cleansing from potential sporadic artifacts were ensured both by visual inspection of the rhythmogram and Poincare plot and by using triangular interpolation of the NN interval histogram with the calculation of the TINN (TINN-2S/AMo). The mathematical algorithm of this index mitigates the impact of artifacts on the final metrics. HRV characteristic time-domain metrics (Mo, AMo, DX, SDNN, RMSSD, pNN50, HRV index, SI, V, VAI) and frequency-domain metrics (VLF, HF, LF_norm_, HF_norm_, LF/HF) were calculated strictly based on the inter-pulse intervals of the PPG signal obtained from the finger sensor. The calculation and interpretation of reference HRV values were performed in accordance with the international guidelines of the Task Force of the European Society of Cardiology and the North American Society of Pacing and Electrophysiology (1996), as well as the Guidelines for the Application of Heart Rate and Heart Rate Variability in Occupational Medicine and Occupational Health Science [25,26,27]. 

HRV recording and primary analysis were performed using the Eldar medical-technical system. The evaluation of frequency-domain and time-domain parameters was based on the integration of international Task Force standards (1996) and domestic guidelines for adaptive potential analysis developed by Baevsky R.M. (2001) [26,27,28] (Table 1).

At the third stage, two machine learning algorithms were used for modeling. Since the analysis incorporated pre-calculated time-domain, geometric, and frequency-domain HRV metrics rather than the raw PPG waveform, this approach represents a feature-engineered model rather than an end-to-end model based on the raw signal.

To assess the additional discriminatory value of HRV parameters obtained using PPG, two sets of predictors were formed:

- Clinical—only clinical-demographic, anthropometric, hemodynamic, and laboratory-functional parameters;
- Clinical_HRV—the same set with the addition of PPG-HRV parameters.

For comparison, two machine learning algorithms were used: logistic regression with elastic net regularization as an interpretable linear model, and random forest as a nonlinear ensemble model.

Before building the models, we controlled for potential target variable leakage. We excluded features from the predictor space that directly reflected an established cardiovascular diagnosis or closely related treatment decisions, including coronary artery disease, chronic heart failure, beta-blocker therapy, and ACE inhibitor/calcium channel blocker therapy. This step was necessary to minimize the risk of artificially inflating classification quality due to target leakage.

All preprocessing was performed strictly within the pipeline, eliminating data leakage between the training and test sets. Most-frequent imputation was used to handle missing values. In the logistic regression model, feature standardization was performed after imputation using StandardScaler, as the linear regularized model is sensitive to the scale of variables. Standardization was not used for random forest, as it is not required for tree-based models. For both models, the class_weight = “balanced” parameter was used to partially compensate for moderate class imbalance.

For logistic regression, the hyperparameter space included the regularization coefficient C = [0.01, 0.1, 1, 10] and the ratio l1_ratio = [0.0, 0.25, 0.5, 0.75, 1.0]. For the random forest, the number of trees n_estimators = [100, 200, 300], the maximum depth max_depth = [None, 3, 5, 7], the minimum number of observations for a node split min_samples_split = [2, 5, 10], and the minimum number of observations in a leaf min_samples_leaf = [1, 2, 4].

Model evaluation was performed using nested cross-validation, which allowed us to separate the hyperparameter selection stage from the final quality assessment. The outer loop used stratified 5-fold cross-validation to evaluate model quality, while the inner loop used stratified 3-fold cross-validation to select optimal hyperparameters. The final metrics were calculated using out-of-fold predicted probabilities, that is, the probabilities for objects not involved in training the corresponding model. This approach was chosen to minimize optimistic bias in the evaluation on a small sample.

To assess the quality of the models, ROC-AUC and PR-AUC were used as discriminatory power metrics, Brier score and LogLoss as probabilistic estimate quality metrics, and accuracy, sensitivity, and specificity were at a fixed classification threshold of 0.5. For the best model, calibration analysis and confusion matrix analysis were additionally performed.

Permutation importance and SHAP were employed to interpret the contribution of features. Permutation importance was calculated as the average reduction in ROC-AUC across 30 random permutations of individual feature values. SHAP analysis using TreeExplainer was applied to interpret the nonlinear Random Forest model, with feature importance estimated using the mean absolute SHAP value.

Date analysis was performed using Python 3.12. Data preprocessing amd manipulation of table structures were performed using the pandas and NumPy libraries. The scikit-learn library was used to develop and validate machine learning models, results were visualized using matplotlib, and the resulting models were interpreted using the SHAP library.

### Statistical Analysis

The statistical analysis of the obtained data was carried out using the SPSS Statistics 22.0 package (IBM, USA). Quantitative data were presented as the median and interquartile range (Me [Q1; Q3]) with a distribution other than normal, and as an average with a standard deviation (M ± SD) for normally distributed data. Nominal variables were represented as absolute and relative frequencies (%). The distribution of qualitative characteristics between the groups was compared using Pearson’s χ^2^-criterion. To conduct intergroup pairwise comparisons of two independent samples, the Shapiro–Wilk test (for a normal distribution) and the Mann–Whitney U-test (for a distribution other than normal) were used, taking a statistically significant level at *p* < 0.05. The differences between the compared groups were considered statistically significant at *p* < 0.05. Spearman correlation was used to assess the relationship between groups and indicators.

Due to the relatively small sample size (*n* = 100) and to minimize the risk of overfitting, the discriminatory ability of the models was assessed using nested cross-validation, with final metrics calculated based on out-of-fold predictions. This approach provided more rigorous internal validation compared to a single split of the sample into training and testing parts.

## 3. Results

According to the results of our study, when comparing the characteristics of the general sample in people with and without CVD, the following data were obtained, presented in Table 2. The average age of men was 80.1 ± 8.7 years, and for women, 81.6 ± 9.0 years. The individuals included in the study were divided into two groups depending on the presence of CVD. Group 1 included 54 participants with CVD who had a history of cardiovascular disease. The average age was 81 ± 9.0 years, men 43% (*n* = 23) and women 57% (*n* = 31). Group 2 included 46 people without CVD and at risk of developing it. The average age was 81 ± 8.4 years, men 40% (*n* = 17) and women 60% (*n* = 29).

In the study, the groups were comparable in age, gender, marital status, and education, with no statistically significant differences (*p* > 0.05). A comparison of the heredity between groups with and without CVD, performed using the χ^2^-Pearson test, revealed statistically significant differences; thus, heredity as a factor was significantly more common in people with CVD (74% vs. 39%, *p* < 0.001).

When studying anthropometric indicators, it was determined that the average BMI in the group with CVD was 26.3 ± 3.1 kg/m^2^ and in the group without CVD it was 22.0 ± 3.0 kg/m^2^, which reflects overweight; abdominal obesity prevails, since the waist circumference in people with CVD reached 103 ± 10.5 cm in men and 96.3 ± 10.8 cm in women, and thus there is a significant statistical difference between the groups. Differences in risk factors such as smoking and alcohol abuse were statistically unreliable, which may be due to the lack of a history of previous episodes of smoking and alcohol use. The analysis revealed a statistically significant decrease in physical activity levels among subjects with CVD: the total duration of continuous physical activity was less than 30 min per day compared to the control group (*p* < 0.05). Blood pressure measurements revealed a statistically significant (*p* < 0.05) increase in systolic blood pressure in subjects with CVD compared to the control group. Heart rate, an indicator of sympathetic reactivity, was a reliable and significant risk factor for the development of CVD (*p* < 0.05).

The data obtained confirm the significant role of hereditary, metabolic and hemodynamic factors in the formation of the risk of developing CVD and can be used to develop preventive and therapeutic strategies.

**Figure 1:**
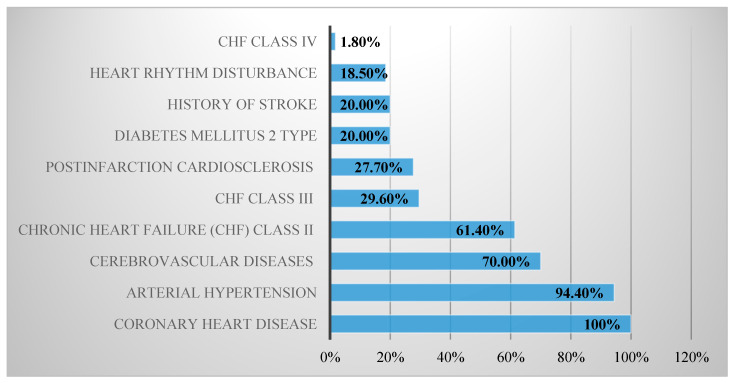
The structure of cardiovascular pathologies in individuals in group 1 (%).

Our analysis showed the diverse prevalence of cardiovascular pathology in patients of the first group. All the examined (100%) had coronary heart disease (CHD), arterial hypertension (AH) was detected in 94.4% of people, chronic heart failure (CHF) of NYHA functional class II in 61.4%, class III in 29.6%, class IV in 1.8%, and cardiac arrhythmia in the form of atrioventricular block and atrial fibrillation were registered in 18.5%. In addition, postinfarction cardiosclerosis (PIC) was detected in 27.7% of the examined patients., diabetes mellitus (DM) in 20%, history of stroke was observed in 20% of people, and cerebrovascular diseases (CVDs) were diagnosed in 70% of individuals (Figure 1). The results obtained indicate a pronounced comorbidity of cardiovascular and cerebrovascular pathology in the study group.

The next step was to analyze the PPG data. Analyzing the data obtained from the study of the PHG index, as can be seen in Table 3, in the group of people with CVD, the time indicators of total heart rate variability revealed a decrease in SDNN by 2 times (26 [Q1–Q3:15, 35] ms), pNN50 by 3.5 times (4 [Q1–Q3: 3, 5]%), TINN (31 [Q1–Q3: 20, 51] ms) by 5 times, and HRV (6 [Q1–Q3: 4, 8.7] ms) by 2.5 times, which is typical for people with CVD and indicates limited adaptive capabilities of the autonomic nervous system. When comparing the time indicators of total heart rate variability (SDNN, pNN50, TINN, HRV), a significant difference was observed between groups with and without CVD (*p* < 0.05). A comparative analysis of the time-domain parameters revealed that the median RMSSD in the group of patients with CVD was 54 [Q1–Q3: 14, 93] ms, while in the group of individuals without CVD it was 27 [Q1–Q3: 13.5, 69] ms. Due to the high heterogeneity of the data and the presence of single extreme values, no statistically significant differences were found between the groups. The spectral indices of VLF ms^2^, LF ms^2^, and HF ms^2^ characterizing cardiovascular dysfunction were significantly reduced in the group of people with CVD, the median was VLF 3510 [Q1–Q3: 2450, 4500] ms^2^, LF 1050 [Q1–Q3: 750, 1500] ms^2^, HF 622.5 [Q1–Q3: 450, 750] ms^2^ (*p* < 0.05). The ratio of VLF ms^2^ to HF ms^2^ power in the first group was 5.84 median [Q1–Q3: 2.48, 10.11] ms^2^, and in the second 2.62 [Q1–Q3: 1.51, 3.77] ms^2^, and when comparing the ratio of VLF/HF between the groups, statistically significant differences were revealed; in people with CVD it was higher compared with the group without CVD (*p* < 0.05). The higher the ratio of VLF to HF, the higher the likelihood of chronic stress and vascular dysfunction. In the analysis, the median LF_norm_ in the CVD group was 59.5 [Q1–Q3: 54, 68], while in the group without CVD it was 52 [Q1–Q3: 48.25, 58.5]; the median HF_norm_ was 38 [Q1–Q3: 28.5, 46] in CVD individuals and 41 [Q1–Q3: 34, 49] in individuals without CVD. These indicators were accompanied by statistically significant differences (*p* < 0.05). Also, the analysis of the LF/HF index reflecting the balance of the sympathetic and parasympathetic parts of the autonomic nervous system showed a significant increase in patients with CVD compared with the group without CVD (*p* = 0.003).

Thus, in patients with CVD, there is a decrease in overall variability and parasympathetic regulation (SDNN, pNN50, TINN, HRV), as well as a marked decrease in HRV spectral parameters (VLF, LF, HF) with a relative increase in the VLF/HF ratio, which indicates a global suppression of autonomic regulation of the heart rhythm and a predominant decrease in the parasympathetic effect. The predominance of sympathetic activity in individuals with CVD (LF_norm_, HF_norm_, LF/HF) has been established. These results of PPG in people without CVD show healthy autonomic regulation of the heart rhythm, and in the future they can serve as a guideline for comparison with patients with CVD.

### Correlation Analysis

The correlation analysis examined the relationship between clinical and demographic indicators and HRV parameters in groups of people with and without CVD. Correlation analysis in individuals with CVD revealed a statistically moderate negative association of indicators of general and parasympathetic HRV with age (SDNN r = −0.52; RMSSD r = −0.48; pNN50 r = −0.45; HRV r = −0.50; TINN r = −0.46; *p* < 0.05) and with systolic blood pressure (SDNN r = −0.44; RMSSD r = −0.41; pNN50 r = −0.40; HRV r = −0.42; TINN r = −0.40; *p* < 0.05) and a moderate positive association with physical activity (SDNN r = 0.42; RMSSD r = 0.40; pNN50 r = 0.38; HRV r = 0.41; TINN r = 0.39; *p* < 0.05). Individuals without CVD had a statistically weak association with age, physical activity, and heart rate. The remaining indicators did not show a significant correlation. Thus, age negatively correlates with all time-related HRV indicators; the older a person is, the lower the variability in humans, and people with CVD experience a more pronounced decrease in HRV with age and an increase in systolic pressure. Physical activity correlates positively with HRV in both groups, but the effect is stronger in CVD patients. Thus, the more physical activity, the higher the HRV indicators, and the lower the physical activity, the lower the HRV parameters.

Analysis of spectral HRV revealed a significant positive relationship between the power in the ultra-low-frequency range of the VLF indicator and the VLF/HF index with age (r = 0.32, r = 0.30; *p* < 0.05), with BMI (r = 0.41, r = 0.31; *p* < 0.05), and with the level of systolic blood pressure (r = 0.36, r = 0.32; *p* < 0.05). This trend may reflect a shift in the autonomic balance towards increased sympathetic and central humoral-metabolic influence on the heart rhythm in older individuals and patients with increased BMI, which indicates a possible increase in sympathetic-adrenal activity.

Indicators of parasympathetic activity showed a significant positive correlation of LF and HF with the level of physical activity (r = 0.34, r = 0.38; *p* < 0.05) and also had a negative correlation with heart rate (r = −0.29, r = −0.33; *p* < 0.05), which may indicate a decrease in vagal influence with increased heart rate. In the group of people without CVD, the structure of correlations was somewhat different. The most pronounced statistically significant correlations were observed between the level of physical activity and LF and HF (r = 0.40, r = 0.42; *p* < 0.05), as well as a negative correlation of heart rate with HF (r = −0.35; *p* < 0.05). In addition, there was a moderate negative relationship between VLF/HF and physical activity (r = −0.31; *p* < 0.05) and a positive relationship with systolic blood pressure (r = 0.27; *p* < 0.05). Thus, the results obtained show that in patients with CVD, the indicators of VLF, LF, HF, VLF/HF are largely associated with metabolic and hemodynamic risk factors, whereas in people without CVD, the level of physical activity plays a more significant role in the formation of vegetative balance.

In individuals with CVD, these correlations of sympathetic-parasympathetic imbalance were more pronounced than in the group without CVD. Correlation analysis showed a different nature of the interrelations between socio-clinical indicators and HRV parameters in the studied groups. In individuals with CVD, there was a moderate positive correlation between age and LF_norm_ (r = 0.53) and LF/HF (r = 0.58), as well as a negative correlation with HF norm (r = −0.53), indicating increased sympathetic influence and decreased parasympathetic activity with age (*p* < 0.05). In the group of people without CVD, the most pronounced associations were between physical activity and HF_norm_ (r = 0.51) and LF/HF (r = −0.58), which indicates a beneficial effect of physical activity on vegetative balance (*p* < 0.05). In addition, a strong correlation was found between HR and LF/HF (r = 0.75, *p* < 0.05). Thus, in the group of patients with CVD, age was the main factor influencing the spectral parameters of HRV, such as LF_norm_, HF_norm_ and LF/HF, with sympathetic predominance and decreased parasympathetic activity. In people without CVD, physical activity and heart rate were the main factors, which means that physical activity increased parasympathetic tone and improved sympathovagal balance. The data obtained emphasize the importance of age and lifestyle in the formation of autonomic control of cardiac activity.

Thus, the results of the study demonstrate that HRV parameters are closely related to the blood pressure, age, and physical status of the examined individuals.

The use of machine learning allowed us to evaluate the additional discriminatory value of models based on a combined set of clinical data and heart rate variability indices obtained using photoplethysmography, compared with models built solely on clinical data. The addition of HRV features derived from PPGs resulted in improved classification performance in both the logistic regression and random forest models. This indicates the additional discriminatory value of HRV features in the task of classifying the presence of CVD. A comparative analysis of the effectiveness of machine learning models is shown in Table 4.

The RF_clinical_HRV model demonstrated the best overall results, for which ROC-AUC was 0.9988, PR-AUC 0.9990, Brier score 0.0279, LogLoss 0.1344, accuracy 0.98, sensitivity 0.9815, and specificity 0.9783. The LR_clinical_HRV model showed almost comparable quality: ROC-AUC 0.9984, PR-AUC 0.9987, accuracy 0.98, sensitivity 0.9630, and specificity 1.0000. Thus, random forest provided the best overall classification quality, while logistic regression remained the most interpretable linear alternative.

Both models based solely on clinical features demonstrated slightly lower results. For LR_clinical, the ROC-AUC was 0.9875, while for RF_clinical, it was 0.9855. After adding HRV features, the performance of both models improved, confirming the consistent additional information provided by photoplethysmographic HRV parameters.

**Figure 2:**
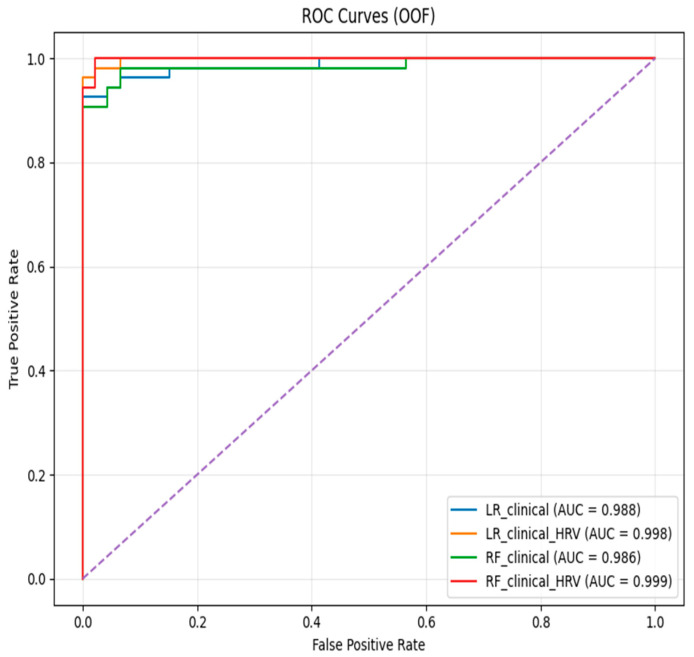
ROC curves of the studied models under nested cross-validation.

The presented ROC curves are based on out-of-fold predictions for all models. All models exhibit high discriminatory power, but both combined models are located closer to the upper left corner of the coordinate plane compared to models built solely on clinical features. This graphically confirms the advantage of combining clinical information with photoplethysmographic HRV features. The results of the comparative models are presented in Figure 2.

**Figure 3:**
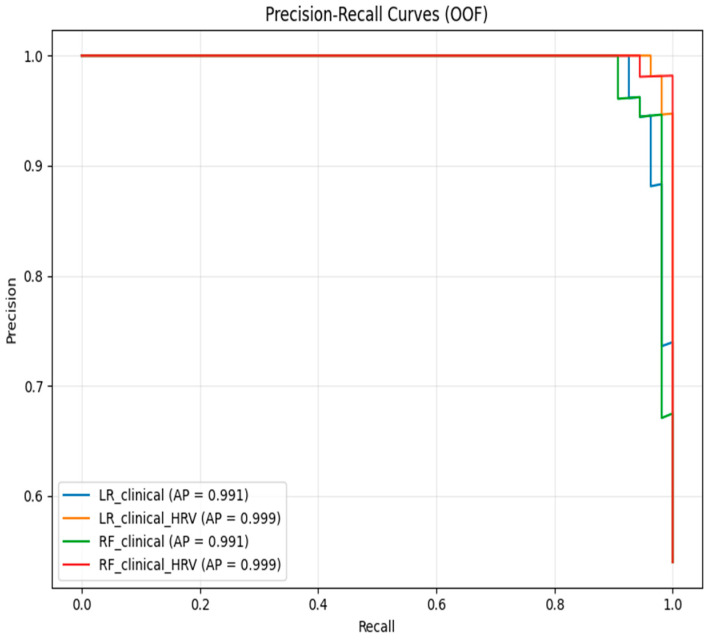
Precision-recall curves of the studied models under nested cross-validation.

Additionally, the robustness of the models was analyzed using precision-recall curves. The comparative analysis results are presented in Figure 3.

Analysis of precision-recall curves demonstrated the same pattern. Models with a combined set of features outperformed models on clinical data, indicating a higher ability to correctly identify the positive class. The best values were obtained for RF_clinical_HRV and LR_clinical_HRV.

For a detailed analysis of classification accuracy at the individual prediction level, a confusion matrix was constructed for the RF_clinical_HRV model. The analysis revealed 45 true negatives, 53 true positives, 1 false positive, and 1 false negative. Thus, the model made only two errors across the entire sample, consistent with its high accuracy, sensitivity, and specificity values.

The SHAP method was used to interpret the decision-making mechanisms of the RF_clinical_HRV model and assess the contribution of each variable at the individual level. Arterial hypertension was the most important variable. Other influential variables included heart failure, physical activity, RMSSD, body mass index, LF, pNN50, LF/HF, and total cholesterol. The SHAP summary plot showed that the model does not rely on a single leading predictor but rather forms a decision by jointly considering clinical factors and autonomic regulation indicators. The results indicate that photoplethysmographic HRV markers do contribute to the differentiation of groups with and without CVD.

**Figure 4:**
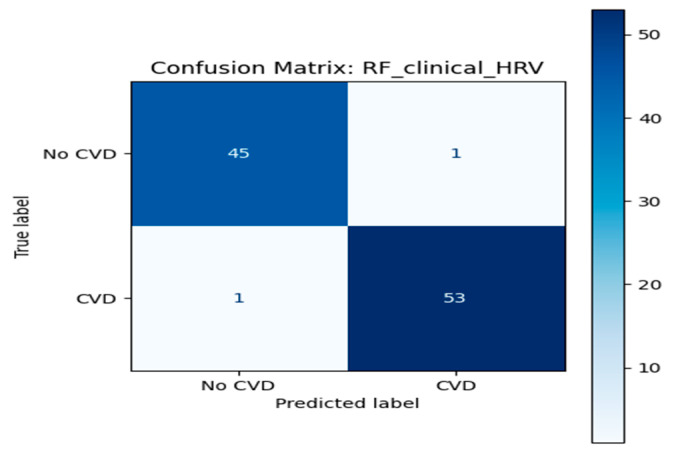
Error matrix of the best random forest model with a combined set of clinical and photoplethysmographic HRV features.

Taken together, the results show that HRV features derived from the PPG method improve CVD classification compared to models built solely on clinical data. The random forest demonstrated the best overall performance, while logistic regression retained its status as the most interpretable and clinically transparent alternative. This analysis is visualized in Figure 4.

The error matrix analysis of the best RF_clinical_HRV model showed high diagnostic accuracy: 45 true negatives, 53 true positives, 1 false positive and 1 false negative. Thus, the model made only two errors in the entire sample of 100 patients, which is consistent with very high values of accuracy, sensitivity, and specificity.

**Figure 5:**
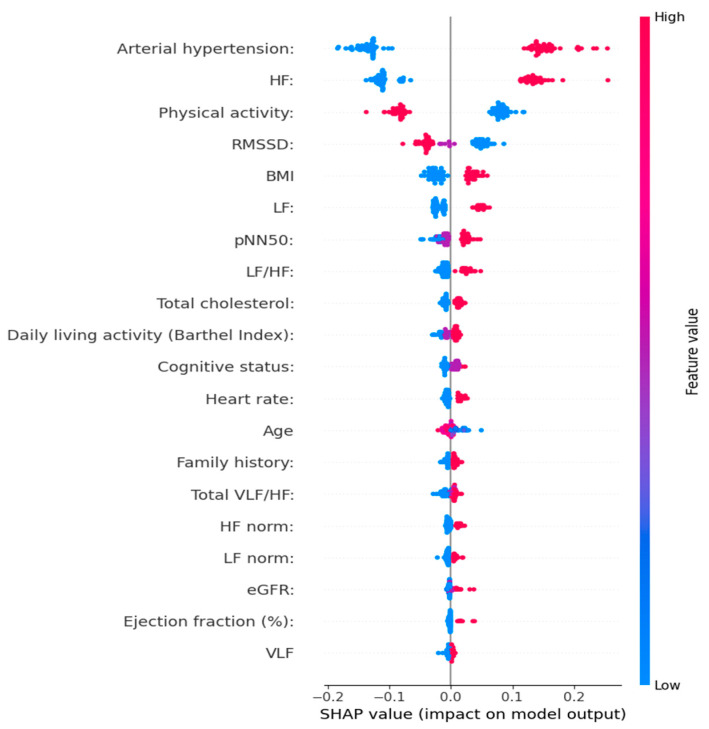
SHAP summary plot for a random forest model with clinical and photoplethysmographic HRV features.

The SHAP method was used to interpret the decision-making mechanisms of the RF_clinical_HRV model and assess the contribution of each feature at an individual level. The results are shown in Figure 5.

Interpretation of the model using the SHAP analysis method allowed for clinical adequacy. Arterial hypertension again turned out to be the most important sign. HF, physical activity, RMSSD, BMI, LF, pNN50, LF/HF, and total cholesterol were also among the most influential variables. The SHAP summary plot showed that the model does not rely on a single leading predictor but forms a solution by taking into account clinical factors and signs of autonomous regulation together. This indicates that photoplethysmographic HRV markers do contribute to the differentiation of groups with and without CVD rather than acting as secondary or random variables. The obtained correlations are generally consistent with the results of the SHAP analysis of the nonlinear model, where age and systolic blood pressure also demonstrated a significant contribution to the classification of current cardiovascular status.

Collectively, the results show that signs of heart rate variability obtained by photoplethysmography improve the classification of cardiovascular diseases compared with models based solely on clinical data. At the same time, the random forest demonstrated the best overall quality, while logistic regression retained the status of the most interpreted and clinically transparent alternative.

## 4. Discussion

Our study investigated the clinical value of HRV indices in the diagnostic prediction of CVD risk stratification. The results showed that individuals with CVD had lower HRV values compared to the group without CVD. The results confirmed that the presence of cardiovascular system pathologies affects HRV. In particular, the decrease in overall variability (SDNN, pNN50, TINN, HRV) and HRV spectral parameters (VLF, LF, HF, LF norm, HF norm, LF/HF) shows a significant relationship with age, physical activity, BMI and systolic blood pressure. While decreased HRV parameters in cardiovascular disease are well known, this study expands the existing understanding through a comprehensive analysis of autonomic regulation in the context of aging. The findings demonstrate that in elderly and senile individuals, decreased HRV reflects not simply a deficit in autonomic influences but is a marker of systemic desynchronization and accelerated aging of the cardiovascular system due to the cumulative effects of modifiable risk factors. Our study is the first in Kazakhstan to adapt HRV-based machine learning models to a specific and highly vulnerable group—the elderly—filling a gap in regional geriatric data.

Thus, the original value of the work lies in the justification of the combined use of HRV parameters and basic clinical and demographic indicators for stratification of the identification of high clinical risk groups at the stage of primary screening.

The results of the study are consistent with the existing literature. Recent studies have shown (2025) that a decrease in HRV parameters is associated with cardiovascular mortality in patients with CHF. A decrease in the SDNN value was considered the most significant predictor of an unfavorable prognosis [32]. Although in prospective studies, HRV parameters demonstrate prognostic significance for the development of CHF and mortality, in our work they are considered as potential digital markers for diagnostic prediction and assessment of the probability of CVD risk.

In studies by Bruno Ferraz et al., it was shown that in patients with heart failure hospitalized for decompensated heart failure, the 10 min RMSSD, age and left ventricular ejection fraction were reduced. These results showed that even short-term activation of sympathetic activity can have an impact on survival, especially in elderly patients with CHF with a reduced fraction [21]. Our study found that with increasing age, there is a general decrease in VSP indicators, with the exception of RMSSD, while a statistically significant relationship was found between age and all HRV indicators.

In the scientific literature of the late 20th century, the SDNN index was the “gold standard” for the stratification of cardiac risk with 24 h HRV registration. SDNN values have been shown to have predictive significance in terms of both morbidity and mortality [33]. The data we obtained on a decrease in HRV rates in the examined individuals are consistent with the results of previous studies. However, further, more in-depth research is required to confirm the diagnostic and discriminatory value of HRV indicators in both patients with CVD and healthy individuals.

A meta-analysis of cohort studies showed (2020) that a decrease in HRV was associated with a higher risk of death from all causes in patients with CVD. The combined risk ratio for death from all causes was 2.27 [95% confidence interval (CI): 1.72, 3.00], and for cardiovascular events it was 1.41 (95% CI: 1.16, 1.72). Thus, HRV shows its promise as a predictor of cardiovascular risk. However, changes in HRV indices were associated with acute myocardial infarction and coronary syndrome and did not reach significance in patients with coronary heart disease and CHF [34]. Our study involved individuals with a history of cardiovascular pathology, outside of an acute condition. There was a significant decrease in HRV in patients with coronary heart disease, CHF and arterial hypertension. Thus, a decrease in HRV parameters is also observed in stable and chronic diseases and can be used for diagnostic prognosis of the current profile and stratification of patients.

The Bo He study revealed a negative correlation between systolic and diastolic blood pressure and HRV (r = −0.50, r = −0.45, *p* < 0.001), as well as SDNN and RMSSD with systolic blood pressure (r = −0.46, *p* < 0.001; r = −0.41, *p* < 0.024). In this study, the authors obtained a loan link between systolic blood pressure and markers of inflammation such as C-reactive protein, TNF-α and IL-1β, and this shows the risk of developing factors in the progression of CVD [22]. Our results are consistent with the data of Bo He and co-authors, which also demonstrated a decrease in SDNN and RMSSD. In our study, in contrast to these studies, an extended analysis of HRV parameters was carried out. Statistically significant correlations have been established between systolic blood pressure and HRV, including SDNN, RMSSD, pNN50, HRV, TINN, VLF, and VLF/HF. In addition, when interpreting the results, key risk factors for CVD development were taken into account, including behavioral, genetic, demographic and functional characteristics, which increases the validity and clinical significance of the data obtained.

Marcos Antonio and co-authors conducted a cross-sectional study in people aged 40 to 100 years with risk factors for developing CVD. In this study, a long-term electrocardiogram was used. The results showed that the parameters SDNN, SDANN, RMSSD, and pNN50 decreased linearly with age and BMI in both sexes; in women, the value was lower than in men (*p* < 0.001). Individuals with diabetes mellitus (DM) were found to have lower levels of HRV, but increased blood pressure and dyslipidemia did not show an effect on HRV reduction [35]. Our work also revealed a decrease in these indicators, which demonstrated a statistically significant correlation with age and BMI. In addition, the relationship of the parameters with an increase in systolic blood pressure has been established.

Anton R Kiselev and co-authors in 2020 studies showed that all spectral parameters of HRV (LF%, HF%, and LF/HF) are quite sensitive and specific for hypertension and coronary heart disease. In this case, the HF index increased two to five times in patients with arterial hypertension and up to eight times in patients with coronary heart disease compared with healthy people. Later, the authors showed that these spectral indicators act as highly sensitive markers of the risk of developing CVD [29,36]. Our data are partially inconsistent with these results. In this study, we analyzed both absolute and normalized HRV values to compare the variability and stability of this parameter. The analysis revealed a significant decrease in the LF% and LF/HF ratio, but the dynamics of normalized HF% units demonstrated the opposite trend. We believe this discrepancy is due not to a true increase in parasympathetic tone, but to the mathematical peculiarities of calculating normalized parameters in the geriatric population.

When interpreting the study results, it is necessary to consider the methodological features of analyzing spectral HRV parameters in individuals over 65 years of age using the PPG method. Unlike younger cohorts, elderly patients with comorbidities and a high medication load exhibit a critical drop in total spectral power (TP). In the geriatric population, the calculated spectral parameters (LF, HF, LF/HF) reflect complex cardiovascular coupling rather than isolated central chronotropic regulation. Under the conditions of the calculation formula, this leads to an artifactual overestimation of the percentage contribution of LFnorm and HFnorm. Thus, the phenomenon we observed is primarily methodological in nature, which confirms the need for a priority analysis of absolute values when assessing the vegetative status in older individuals [37,38,39,40,41,42].

Shujuan Liu et al. (2025) [23], in their scientific review, reported a decrease in SDNN and RMSSD in CHD, DM, CHF, depression and CKD, RMSSD in obesity and epilepsy, LF in depression and Parkinson’s disease, HF in CHD, depression, obesity and epilepsy, and increased LF/HF in CHD, depression and obesity. The authors state that HRV remains a promising but not yet confirmed biomarker, since the value of HRV indicators has not reached the threshold of evidence for clinical decision-making. In this work, in accordance with the data presented in the scientific review by Shujuan Liu, a decrease in HRV and an increase in the ratio of LF/HF with CVD were revealed. Our results also support the importance of further investigation of HRV parameters as potential markers for CVD stratification.

Since 2000, the relationship between age and gender with activation of the autonomic system has been investigated. A total of 114 men and 75 women participated in the Hulegar A. Abhishekh studies, with a 5 min electrocardiogram used for the study. The results showed that the indicators SDNN, RMSSD, HF, and LF/HF correlated with age (SDNN: r = −0.444; RMSSD: r = −0.552, HF: r = −0.167; LF/HF r = 0.19; *p* < 0.01;). In conclusion, the authors revealed a predominance of sympathetic and a decrease in parasympathetic tone with age; in men, vagal tone was lower than in women [43]. A recent study using PHG in healthy Asian adults showed (2020) that all HRV parameters decrease with age in the Asian population (*p* < 0.001 for all indicators), as observed in the Western population [44]. In a cross-sectional study, Aravind Natarajan and co-authors analyzed HRV rates measured with FPG in 8 million people. The study found that HRV parameters decrease with age, while parasympathetic function deteriorates faster than sympathetic function. The researchers also noted that physical activity improves HRV in healthy people [45]. Currently, the study of risk factors for the development of cardiovascular pathologies in older people is relevant, since gender and age are some of the most important unmodified risk factors. According to the scientific literature, differences in CVD risk between men and women diminish with age. While age- and gender-specific factors are traditionally included in most prospective models, they are necessary for diagnostic prognosis and assessment of the likelihood of CVD in cross-sectional studies [46,47,48]. Our study also demonstrates an age-associated decrease in HRV, with a decrease in variability with increasing age. However, there was no statistically significant relationship with gender. In individuals with CVD, the decrease in HRV was more pronounced, whereas participants without CVD maintained relatively favorable HRV values regardless of age. The findings emphasize the need for further HRV research in the context of cardiovascular pathology.

In the study of Lin IM, Lin PY and Fan SY. The effect of HRV biofeedback using baroreflex on HRV recovery in individuals with coronary heart disease under conditions of emotional stress was studied. According to the results, the HRV biofeedback showed good HRV indicated increased parasympathetic regulation and better adaptation to stress. The data may be important for the prevention of adverse cardiovascular events [49]. The limitations of the study include a relatively small sample of participants, the short-term nature of the observation and the lack of assessment of the impact of concomitant factors. In general, it is emphasized that further research is needed to clarify the role of HRV in the development and risk assessment of CVD, as well as to determine its preventive value.

Many studies claim that HRV research is a promising digital marker for identifying cardiovascular risk and monitoring treatment, disease course, and prognosis, and the authors claim the need for the standardization of protocols and large-scale research to establish the exact value of HRV using PHG [22,50,51]. There are a number of studies on the effect of inflammatory markers on HRV affecting the function of the autonomic nervous system; these data prove the importance of HRV as a potential digital marker for predicting cardiovascular inflammation and the diagnostic predicting of CVD [22,50,51,52].

Despite the results obtained, the indicated study has a number of limitations. Firstly, the study was cross-sectional in nature, which does not allow us to assess the causal relationship between age, clinical factors, and changes in HRV (Almeida-Santos et al.; Choi et al.). Secondly, the studies did not take into account some potentially influencing factors, such as drug therapy, stress levels, socio-demographic characteristics, and metabolic factors. Thirdly, most of the studies conducted in the age categories are retrospective; there were restrictions on the use of PHG in the elderly. In addition, not all studies conducted comparative analyses with and without CVD risks [35,43,44,45,53,54,55].

From a machine learning perspective, the most important finding of the study is that adding HRV features derived from photoplethysmography improved both models compared to baseline models based solely on clinical data. This effect was observed for both logistic regression and random forest, reducing the likelihood that the added value of HRV is due to the specificities of only one algorithm.

The best overall classification performance was demonstrated by a random forest with a combined set of clinical and HRV features, while logistic regression maintained virtually comparable performance and provided greater interpretability. This result is methodologically important, as it demonstrates that PPG-derived HRV features can act as an additional source of discriminatory information in the task of cross-sectional classification of the presence of CVD in older adults.

SHAP analysis confirmed that the model’s decision making is not based on a single dominant predictor but rather by jointly considering clinical factors and autonomic regulatory factors. The most important variables were hypertension, heart failure, physical activity, RMSSD, body mass index, LF, pNN50, and LF/HF. This allows photoplethysmographic HRV features to be viewed not as random additional parameters but as features that genuinely improve the discrimination between groups with and without CVD within the classification model used.

However, the presented models should be interpreted as a tool for the one-time classification of current cardiovascular status, not as a means of predicting future cardiovascular events. Therefore, the obtained results reflect the additional discriminatory value of HRV features within a cross-sectional study and should not be interpreted as evidence of long-term predictive ability.

The strength of the study is not only the high discriminative ability of the models but also an expanded approach to their evaluation. The work considered not only ROC-AUC and PR-AUC, but also Brier score, LogLoss, calibration analysis, error matrix, permutation importance and SHAP. This approach is fundamentally important because it allows us to evaluate the model not only as a class separation tool but also as a system that forms probabilistic decisions that must be clinically meaningful and interpretable. An additional advantage is the use of nested cross-validation, which reduces the risk of overestimation of quality in a small sample.

Nevertheless, the results obtained should be interpreted with caution. Firstly, the study was performed on a relatively small sample, and even with strict internal validation, a certain degree of optimism in the results cannot be excluded. Secondly, there was no independent external validation, so conclusions about the portability of the model to other populations are still limited. Thirdly, the work analyzed not raw photoplethysmographic signals but derived signs of heart rate variability. This was a conscious choice in favor of interpretability and robustness in a limited sample; however, it means that the study should be considered as a feature-based ML framework rather than an end-to-end raw signal solution. Finally, this study addresses the problem of cross-sectional classification of the presence of cardiovascular disease rather than the problem of long-term prediction of clinical outcomes.

Thus, age-related changes in the vagal system are considered as a predisposing factor for the development of CVD and a promising digital marker, but HRV research has not yet reached the level of diagnostic and prognostic values. Currently, there is no regulatory data for short-term HRV measurements, which requires confirmation in prospective and large-scale population studies covering the entire age spectrum.

### Limitations of the Study

The main limitation of this study is the relatively small sample size (*n* = 100: 54 participants with CVD and 46 without), which was collected as part of the regional component of a state research project. The cross-sectional design focused solely on modeling current discriminatory power. This study is a pilot analysis within the framework of a state project, and subsequent multicenter studies involving patient cohorts from other regions of the Republic of Kazakhstan are required to confirm the scalability of the developed CVD classification algorithms.

The sample size is relatively small for multivariate analysis using machine learning, increasing the risk of instability in quality estimates and requiring cautious interpretation of the results. Despite the use of nested cross-validation and the execution of preprocessing strictly within the pipeline, a certain degree of optimism in internal estimates cannot be completely ruled out. The models were not externally validated, and the transferability of the findings to other populations and clinical settings remains unknown. This is especially important given that participants were recruited from a limited geographic region and specific medical sites. Furthermore, the work did not use raw photoplethysmographic signals but derived features of heart rate variability. Therefore, this research should be considered as a feature-based ML framework and not as an end-to-end raw-signal AI model.

Key methodological limitations of the study include the potential risk of residual confounding. Autonomic regulation of the heart and the extracted HRV parameters are highly sensitive to somatic background, comorbidity, medication use, and psychoemotional status, necessitating caution when interpreting the results of a cross-sectional analysis.

Firstly, drug therapy, especially the use of beta-blockers and antiarrhythmic drugs that modify chronotropic function, significantly influences the time-domain and frequency-domain parameters of HRV. Currently, there is a lack of original studies and systematic reviews devoted to the effect of antihypertensive, antiarrhythmic and other medications on HRV. Nevertheless, there are studies over the past two decades on the effect of drugs on HRV. The most pronounced increase in HRV was observed with the use of beta-blockers and non-dihydropyridine calcium channel blockers, while ACE inhibitors and diuretics were characterized by a less pronounced effect [56,57,58,59,60]. In our sample, about 30% of patients in the CVD group did not receive regular background therapy at the time, which could have independently contributed to the observed generalized decrease in HRV parameters in this group. However, this study did not conduct a separate analysis of the effect of specific medications on HRV parameters.

Secondly, heart rate disturbances and arrhythmias are traditionally significant confounding factors. In this study, 18.2% of patients in the CVD group had arrhythmias, which required rigorous mathematical filtering of PPG intervals, with the removal of non-sinus cycles to minimize distortions in spectral analysis.

Thirdly, comorbid diabetes and chronic kidney disease can induce cardiovascular autonomic neuropathy [61,62,63]. The lack of in-depth patient stratification by these parameters limits the ability to isolate the impact of CVD on HRV parameters. However, it is worth noting that concomitant somatic conditions in the study cohort were well-controlled. These identified limitations provide guidance for future studies with more stringent participant selection criteria.

Finally, factors such as sleep disturbance, depression, and anxiety were not assessed due to the specific nature of this study. Assessment of participants’ physical activity levels was limited to a brief screening questionnaire, which recorded a marked decrease in the daily duration of uninterrupted exercise (less than 30 min per day) in the CVD group (*p* > 0.05). However, the lack of a direct objective assessment of physical fitness precludes a complete isolation of the e1 contribution to the vegetative status of older adults. Due to the relatively small sample size (*n* = 100) and the pilot nature of the study, detailed multivariate stratification by all the aforementioned somatic and medication-related characteristics was impossible without a critical loss of statistical power and a high risk of overfitting the discriminatory algorithms. The developed approach is positioned as a tool for primary express screening, while strict multicenter monitoring and the isolation of the influence of all listed factors, including depression, sleep quality, and comorbid therapy, are a priority vector for subsequent stages of the implementation of this state scientific project.

Taking into account the above factors, as well as the cross-sectional design of the study, the identified changes in spectral and temporal parameters should be interpreted exclusively as complex phenotypic markers statistically associated with the current clinical status of patients.

However, these features of the PPG method do not negate its clinical value. On the contrary, since the development of CVD in the geriatric population is inextricably linked to arterial remodeling, the ability of PPG analysis to record the integrated hemodynamic response enhances its surrogate value for screening and classifying CVD prevalence in individuals aged 65 years and older. Thus, in our study, the PPG method demonstrated adequate potential as a tool for rapidly assessing the probability of pathology, although the isolated pathophysiological interpretation of its spectral components requires caution. Evaluating the isolated impact of pharmacotherapy on HRV parameters in cohort studies represents a promising direction for future research.

## 5. Conclusions

The obtained data demonstrate a close association between the risk of developing cardiovascular disease and autonomic nervous system dysfunction. The decrease in heart rate variability is most pronounced in elderly individuals with existing cardiovascular disease and can be considered a potential tool for developing diagnostic, prognostic, and risk stratification strategies. The use of machine learning demonstrated that heart rate variability features obtained using photoplethysmography improve diagnostic prognostication and classification of cardiovascular diseases compared to models based solely on clinical data.

## Figures and Tables

**Table 1:** Reference and interpretive metrics of heart rate variability derived from PPG for assessment of adaptive potential [20,27,28,29,30,31].

**Indicators of Heart Rate Variability in the Time Domain**		
**Parameters**	**Standard**	**Meaning**
Standard Deviation of NN intervals (SDNN), ms	≤50—low level 50–100—moderate decrease >100—normal level	Characterizes the overall HRV and the total effect of the sympathetic and parasympathetic nervous system
Root Mean Square of Successive Differences (RMSSD), ms	≤20—low level 20–30—average level >30—normal level	Reflects short-term HRV and predominantly parasympathetic activity
pNN50, %	≤5—low level 5–10—moderate decrease >10—normal level	An indicator of short-term HRV reflecting parasympathetic activity
Triangular Interpolation of NN interval histogram (TINN), ms	≤60—low level 60–100—normal value	Geometric HRV indicator, characterizing the overall variability of RR intervals
**Nonlinear indicators of heart rate variability**		
Very low frequency (VLF), ms^2^	<5000—low level ≥5000—normal range	Reflects slow neurohumoral and metabolic mechanisms of heart rate regulation
Low frequency (LF), ms^2^	<1000—low level 1000–2500—normal value >2500 ms^2^—increased activity	Reflects the mixed influence of the sympathetic and parasympathetic systems
High frequency (HF), ms^2^	<1000—low level ≥1000—normal value	Reflects parasympathetic activity
VLF/HF	≤2—normal balance 2–3—average level >3—chronic stress	Shows long-term sympatho-parasympathetic balance
LFnorm, %	≤30%—normal level 30–60%—balanced level >60%—increased sympathetic activity	Characterizes relative sympathetic activity
HFnorm, %	<30%—decreased parasympathetic tone 30–60%—balanced value >60%—high parasympathetic influence	Characterizes relative parasympathetic activity
Ratio LF/HF	0.5–2.0—normal level >2—sympathovagal balance	Shows short-term sympatho-parasympathetic balance

**Table 2:** Socio-demographic and clinical characteristics of the studied groups.

Indicators		Group 1 with CVD (*n* = 54)	Group 2 Without CVD (*n* = 46)	*p*-Level
Age (year, %)		81 ± 9.0	81 ± 8.4	0.833
Gender	male	43% (*n* = 23)	40% (*n* = 17)	0.856
	female	57% (*n* = 31)	60% (*n* = 29)	0.362
Heredity (%)		74% (*n* = 40)	39% (*n* = 18)	0.001
Waist Circumference (cm)	male	103 ± 10.5	84.2 ± 10.4	0.001
	female	96.3 ± 11.0	83.4 ± 10.2	0.001
Body mass index (M ± m)		26.3 ± 3.1	22.0 ± 3.0	0.001
Smoking (abs. number/%)		32% (*n* = 18)	17.3% (*n* = 8)	0.113
Alcohol abuse (abs. number/%)		14.2% (*n* = 8)	8.6% (*n* = 4)	1.000
Low physical activity < 30 min (M ± m)		94% (*n* = 51)	15% (*n* = 7)	0.001
Systolic blood pressure (M ± m)		140.0 ± 10.0	123.0 ± 9.0	0.001
Diastolic blood pressure (M ± m)		85 ± 6.3	80 ± 6.1	0.001
Heart rate, beats/min (M ± m)		81 ± 6.5	73.6 ± 7	0.001

Note: *p*—reliability of the difference in indicators between the groups (*p* < 0.05).

**Table 3:** Analysis of the results of PPG in people with and without CVD.

Parameters	Group 1 with CVD (*n* = 54)	Group 2 Without CVD (*n* = 46)	*p* Level Manna–Whitney (*p* < 0.05)
SDNN mc	26 [Q1–Q3:15, 35]	50 [Q1–Q3:15, 55]	0.001
RMSSD mc	54 [Q1–Q3: 14, 93]	27.5 [Q1–Q3: 13.5, 69]	0.051
pNN50 (%)	4 [Q1–Q3: 3, 5]	15 [Q1–Q3: 10, 70]	0.001
HRV mc	6 [Q1–Q3: 4, 8.7]	19 [Q1–Q3: 15, 26]	0.001
TINN mc	31 [Q1–Q3: 20, 51]	175 [Q1–Q3: 198, 200]	0.001
VLF mc^2^	3510 [Q1–Q3: 2450, 4500]	5378 [Q1–Q3: 2450, 13,000]	0.001
LF mc^2^	1050 [Q1–Q3: 750, 1500]	4650 [Q1–Q3: 1779, 7800]	0.001
HF mc^2^	622.5 [Q1–Q3: 450, 750]	3205 [Q1–Q3: 1244, 5500]	0.001
VLF/HF	5.84 [Q1–Q3: 2.48, 10.11]	2.62 [Q1–Q3: 1.51, 3.77]	0.000
LF norm (%)	59.5 [Q1–Q3: 54, 68]	52 [Q1–Q3: 48.25, 58.5]	0.000
HF norm (%)	38 [Q1–Q3: 28.5, 46]	41 [Q1–Q3: 34, 49]	0.021
LF/HF	1.65 [Q1–Q3: 1.17, 2.21]	1.24 [Q1–Q3: 1.05, 1.62]	0.003

Note: Me(Q1, Q3): The median is the median with the lower and upper quartiles.

**Table 4:** Comparative effectiveness of machine learning models in nested cross-LR (logistic regression).

Model	ROC-AUC	PR-AUC	Brier Score	LogLoss	Accuracy	Sensitivity	Specificity
RF_clinical_HRV	0.9988	0.9990	0.0279	0.1344	0.98	0.9815	0.9783
LR_clinical_HRV	0.9984	0.9987	0.0404	0.1784	0.98	0.9630	1.0000
LR_clinical	0.9875	0.9913	0.0426	0.1580	0.95	0.9630	0.9348
RF_clinical	0.9855	0.9906	0.0476	0.1952	0.95	0.9444	0.9565

## Data Availability

Data are unavailable due to privacy or ethical restrictions.
